# Low doses of cannabis extract ameliorate non-motor symptoms of Parkinson’s disease patients: a case series

**DOI:** 10.3389/fnhum.2024.1466438

**Published:** 2025-02-24

**Authors:** Ana Carolina Ruver-Martins, Ingrid Andrea Rodriguez Martinez, Vinicius Giesel Holla, Beatriz Larentis de Souza, Elton Gomes da Silva, Diana Marcela Aragon Novoa, Eduardo Souza-Silva, Fabiano Soares Araujo, Rui Daniel Prediger, Francisney Pinto do Nascimento

**Affiliations:** ^1^Laboratory of Medical Cannabis and Psychedelic Science, Universidade Federal da Integração Latino-Americana, Foz do Iguaçu, Brazil; ^2^Departamento de Farmacia, Universidad Nacional de Colombia, Bogotá, Colombia; ^3^Department of Pharmacology, Universidade Federal de Santa Catarina, Florianópolis, Brazil; ^4^Laboratório Reaja, Curitiba, Brazil

**Keywords:** cannabis, THC, CBD, Parkinson’s disease, cognition, insomnia, sleepiness, case series

## Abstract

**Introduction:**

Parkinson’s disease (PD) is mainly characterized by motor symptoms including muscle rigidity, resting tremor and bradykinesia. However, the management of the non-motor symptoms represent a relevant clinical challenger in PD. These non-motor symptoms include cognitive and sleep disturbance and there is evidence that cannabinoids may represent alternative and effective treatments for non-motor symptoms of PD.

**Methods:**

Therefore, this study addressed the effects of oral treatment with cannabis extract on cognition, insomnia, and daytime sleepiness in six patients with moderate PD. The patients were randomized to receive two different doses of a cannabis extract: THC:CBD 250:28 μg/day (*n* = 3) or 1000:112 μg/day (*n* = 3). The assessment of cannabis administration related to the cognitive field was measured by the Montreal Cognitive Assessment test (MoCA test), the insomnia was assessed by the Insomnia Severity Index (ISI), and daytime sleepiness was assessed using the Epworth sleepiness scale (ESS). All clinical evaluations were performed before treatment and at 15, 30, 60, and 90 days of treatment.

**Results:**

The statistical analysis indicated a significant benefit of the cannabis extract treatment, at dose of 1000:112 μg/day after 60 days of treatment, on insomnia assessed by ISI. Moreover, the statistical analysis of data from ISI and MoCA tests showed a trend toward improvement over time, while no significant effect was observed in the ESS. There were no reports of significant adverse effects during the cannabis extract treatment.

**Discussion:**

These results demonstrate benefits of short-time treatment (60 days) with low doses of cannabis extract on insomnia in PD patients. This study provide novel findings of the potential of combining CBD and THC as safe and effective treatments for non-motor symptoms of PD.

## Introduction

Parkinson’s disease (PD) is a neurodegenerative disease characterized by the progressive loss of dopaminergic neurons in the substantia nigra pars compacta (SNc) and the appearance of Lewy bodies, mainly composed by the accumulation of insoluble aggregates of *α*-synuclein protein (Braak et al., 2004). Parkinson’s disease (PD), the second most common neurological disorder among the elderly worldwide, affects approximately 0.3% of the global population, with a prevalence of 1–3% in individuals over 60 years old (Tysnes and Storstein, 2017). In Brazil, a research revealed a prevalence of 3.3% among the elderly, significantly contributing to morbidity and mortality in this age group (Bovolenta and Felício, 2016). Projections indicate that PD will affect 17 million people by 2040, primarily men, with no significant differences between races or ethnic groups, and aging being the most significant risk factor (Dorsey et al., 2018).

The classical motor symptoms include bradykinesia, rigidity and resting tremors. However, PD patients also suffer from diverse non-motor symptoms that encompass sensory abnormalities, cognitive impairments, sleep disturbances, autonomic dysfunction, anxiety and depression (Pfeiffer, 2016; Chaudhuri et al., 2006). For instance, insomnia has been reported in up to 80% of PD patients (Wallace et al., 2020), and its neurobiological mechanisms have been associated to neurodegenerative process in the brainstem, thalamocortical pathways, and ventral tegmental areas in the midbrain leading to sleep–wake disturbances (Wallace et al., 2020). Moreover, conflicting findings on the prevalence of excessive daytime sleepiness (EDS) in PD have been described. Clinical detection and determination of EDS intensity are closely linked to subjective criteria. Additionally, some studies have described EDS as a sleep abnormality related to motor impairments of advanced stages of PD (Mantovani et al., 2018).

Indeed, cognitive impairment is one of the most important non-motor symptoms, experience by 24 to 54% of newly diagnosed PD patients (disease duration <1 year) (Darweesh et al., 2017; Weintraub et al., 2017). Cognitive impairment can present before or at the time of PD diagnosis and gradually worsens as the disease progresses (Weintraub et al., 2017; Aarsland et al., 2021). Cognitive decline impacts PD patients’ activity of daily living and quality of life, thus causing a significant increase in the caregiver’s burden (Leroi et al., 2012). Considering the importance of sleep and cognitive dysfunctions in PD patients, it is critical to identify new pharmacological alternatives to management these non-motor symptoms.

The diagnosis of PD is clinical, based on the exclusion of other causes of parkinsonism and the presence of cardinal symptoms. The current available therapies are symptomatic, aiming to improve quality of life by restoring dopaminergic neurotransmission through different mechanisms. However, the drug efficacy is gradually lost and the current drugs do not alleviate the non-motor symptoms and the progressive dopaminergic neuron degeneration in PD patients. In this context, phytocannabinoids, such as cannabidiol (CBD) and delta-9-tetrahydrocannabinol (THC), emerge as promising agents to improve motor and non-motor symptoms of PD (Babayeva et al., 2016; Lotan et al., 2014; Crippa et al., 2019).

Clinical studies on the use of cannabinoids in the treatment of PD have shown varied results. One clinical trial, despite failed to demonstrate relief of dyskinesia with THC compared to placebo, demonstrated improvement in non-motor symptoms of PD patients (Carroll et al., 2004). Other clinical studies showed that CBD can reduce psychotic symptoms (Zuardi et al., 2009) and improve sleep quality (Chagas et al., 2014) in PD patients. Additionally, it has been demonstrated that cannabinoids can reduce both motor and non-motor symptoms, highlighting their potential as a multimodal therapy (Peball et al., 2020). More recently, researchers conducted a clinical study with PD patients, which reinforced the variability of outcomes, indicating that despite some benefits, it is still not possible to establish a definitive therapeutic recommendation for the use of cannabinoids in PD (Liu et al., 2024). Despite this inconclusive findings obtained so far from clinical studies, it is important to emphasize that many PD patients around the world are under treatment with CBD in combination with THC, and there are some reports showing a greater benefit from the cannabinoids combination than CBD alone in PD (Feeney et al., 2021; Holden et al., 2022).

Therefore, additional research is needed to define what cannabinoids and dose are useful in PD treatment.

### Case presentation

The present study consisted of a series of cases with six participants. The study followed the recommendations by the Brazilian Ministry of Health, under approval by the Human Research Ethics Committee under protocol number CAAE 87333418.9.0000.5515, and Informed consent through the Declaration of Helsinki. All patients were volunteers, and they signed the Free and Informed Consent Term (ICF). Eligibility required idiopathic PD with a score of >20 on the motor, part III, of the Movement Disorder Society Unified Parkinson’s Disease Rating Scale (MDS-UPDRS) (Goetz et al., 2008). Because we sought to study effects of study drug on persons with PD receiving usual medical care, if participants were anti-PD medication, this score was required when the medications were working optimally (i.e., best on state).

The Hoehn and Yahr scale, which is considered the reference standard for PD staging measures, was taken into account as a requirement at the time of recruitment (Goetz et al., 2004). Thus, patients classified as stages III and IV according to this scale were included, as can be seen in Table 1. Other inclusion criteria were age over 40 years and being on stable treatment with levodopa for at least 30 days. Individuals who did not accomplish the above criteria were excluded, as well as those patients who suffered from schizophrenia and/or psychosis beyond PD. Patients who had a history of first-degree relatives with these comorbidities suffered epilepsy, or were Cannabis or other illegal psychoactive substances users in any form of administration were also excluded.

**Table 1:** Clinical characteristics of Parkinson’s disease patients treated with Cannabis extracts.

	Pacient	Age and sex	Parkinson stage (Hoehn and Yahr scale)	Disease duration (years)	Education level	Symptoms in order of intensity from highest to lowest	Current pharmacotherapy
Group 1 THC/CBD (250/28 μg/ml)	1	67 W	3	10	5 years	Stiffness, bradykinesia and postural instability	Levodopa+benzerazide (200/50 mg); Amantadine 100 mg; Pramipexole 1 mg.
	2	76 M	3	10	0	Tremor, bradykinesia and stiffness	Levodopa+benzerazide(200/50 mg); Biperiden 2 mg; Clonazepam 2,5 mg/mL.
	3	74 M	4	10	5 years	Tremor, bradykinesia, postural rigidity and instability	Levodopa+benzerazide(200/50 mg); Pramipexole 0,750 mg; Levothyroxine 75 mg.
Group 2 THC/CBD (1,000/112 μg/ml)	4	49 M	3	9	4 years	Bradykinesia, stiffness and tremor	Levodopa+benzerazide(200/50 mg); Pramipexole 1 mg; Amantadine 100 mg.
	5	55 M	4	12	Completed high school	Bradykinesia, stiffness, tremor, postural instability	Levodopa+benzerazide(200/50 mg); Levodopa HBS and dispersible; Amantadine 100 mg; Entacapone 200 mg; Selegiline 5 mg; amitriptyline 25 mg.
	6	60 W	3	18	Completed high school	Stiffness bradykinesia e tremor	Levodopa+benzerazide(100/25); Pramipexole 0,750 mg; Amantadine 100 mg; Trazodone 150 mg.

W, woman; M, man.

The participants selected for this study were four men and two women aged between 49 and 76 years. They were diagnosed with PD at least 5 years ago, all Brazilian, married, with at least one child, retired, living in urban areas, non-smokers, non-drinkers and without neurological comorbidities. The medications in use are listed in Table 1. According to the severity of the symptoms, they were classified into stages III and IV, which represents a transition between the moderate and severe PD state. It was considered that the level of education due to this factor will be relevant at the time of cognitive assessments.

**Figure 1:**
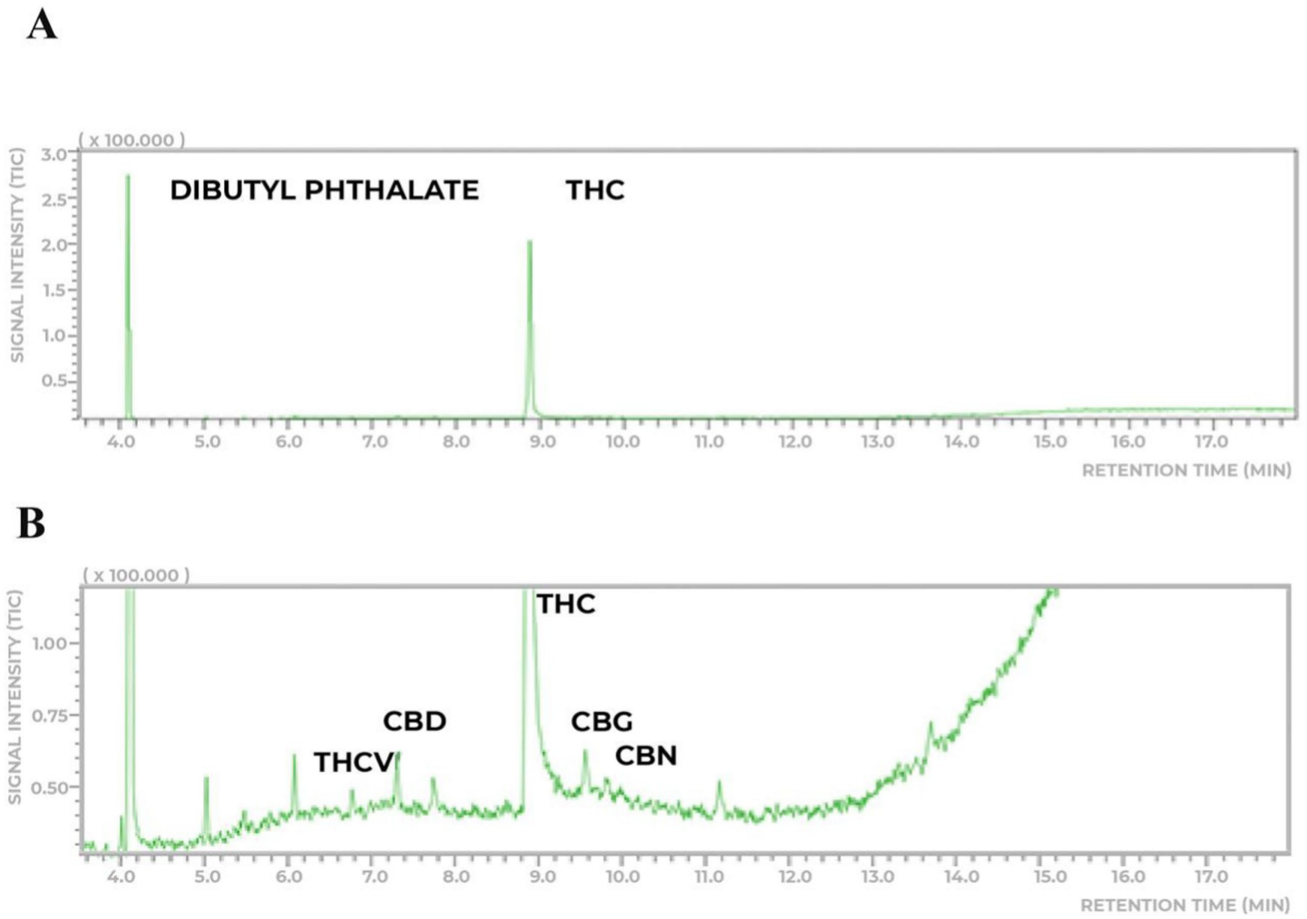
Chemical analyses of the THC-rich cannabis extract. **(A)** The extract analyzed has a higher prevalence of THC (8.7% ± 0.5 m/m) and lower concentration (0.75% ± 0.15) of CBD. **(B)** Thepresence of other phytocannabinoids to a very lesser extent was detected.

The eligible six patients were randomized 1:1 to study the Cannabis extract doses of THC:CBD dose (250:28 μg/day) (group 1) or THC:CBD (1000:112 μg/day) (group 2), by a computer-generated randomization schedule. The cannabis extract used in the study was donated by one of the participants that obtained from a Brazilian Patients Association with legal authorization to plant and develop extracts from cannabis to medicinal use. The cannabis extract was obtained through an artisanal process from a single wild plant. The extraction method involved hydroalcoholic maceration, followed by dilution in olive oil to obtain a full-spectrum oil. The extract was analyzed using gas chromatography coupled with mass spectrometry (GC–MS/MS) on a Shimadzu 2010 with a TQ8040 mass spectrometer and an Rtx-5MS column (30 m × 0.25 mm × 0.25 μm, 5% diphenyl/95% dimethylpolysiloxane). The carrier gas was helium (purity 5.0), and the injection was in split mode (1:90) at 250°C. The temperature program started at 200°C, ramped at 10°C/min to 250°C (held for 6 min), then increased to 280°C at 10°C/min (held for 3 min). The total run time was 18 min. Mass detection was done in SCAN mode (60–400 m/z) with electron ionization at 70 eV. The sample preparation involved dissolving approximately 20 mg of the extract in 8 mL of methanol, followed by magnetic stirring for 10 min. The solution was then filtered to remove any particulates and transferred to a 10 mL volumetric flask, where the volume was adjusted with methanol. For analysis, 0.100 mL of the prepared sample solution was combined with 0.50 mL of internal standard (dibutyl phthalate, 200 μg/mL) (Farzanehfar et al., 2017) and 0.40 mL of methanol, ensuring proper dilution for chromatographic analysis. The sample showed a predominance of THC (8.7% ± 0.5, ~87 mg/mL) with a lower concentration of CBD (0.75% ± 0.15, ~7.5 mg/mL), maintaining a THC:CBD ratio of approximately 9:1 (Figure 1).

The randomization model stratified participants by age and Hoehn and Yahrd score, with three patients being assigned to each treatment group. The participants self-administered 0.5 mL of the respective extract once a day, and the extract was stored at room temperature, protected from light. These doses were based on a previous case report which reflects the clinical decisions in the attempt of finding an optimal dose as a benchmark (Ruver-Martins et al., 2022). All patients administered the Cannabis extract over a period of 90 days. No one drug was added or removed. All patients were taken Prolopa® (levodopa 200 mg + benserazide 50 mg).

We evaluated the cognitive clinical conditions, insomnia, and daytime sleepiness before the beginning of Cannabis extract treatment (T0). The same evaluations were performed at 15 (T1), 30 (T2), 60 (T3), and 90 (T4) days after the beginning of the treatment.

The assessment of cannabis administration related to the cognitive field was measured by the Montreal Cognitive Assessment test (MoCA test). It had 30 questions, in which aspects: of attention and concentration, executive functions, memory, language, visual-constructive skills, conceptualization, calculation, and orientation were taken into account to have a wide scope. A scoring system between 0 and 30 was used, and each success was added to the general score. Values above 26 were considered cognitively normal. The Education level factor was a variable that directly affected the global results (Nasreddine et al., 2005).

The insomnia was assessed by the Insomnia Severity Index (ISI), which consists of seven items that evaluate: (a) the severity of sleep-onset (initial), (b) sleep maintenance (middle), (c) early morning awakening (terminal) problems, (d) satisfaction with current sleep pattern, (e) interference with daily functioning, (f) noticeability of impairment attributed to the sleep problem, and (g) level of distress caused by the sleep problem (Bastien et al., 2001).

Finally, daytime sleepiness was assessed using the Epworth sleepiness scale (ESS) which determines the severity of sleepiness by the likelihood of you being drowsy or falling asleep, and not just tired, in situations such as sitting and reading, watching TV, sitting, being quiet, in a public place, riding in a car for an hour without stopping, as a passenger, sitting quietly after lunch without alcohol, in a car stopped in traffic for a few minutes (Bertolazi et al., 2009).

**Figure 2:**
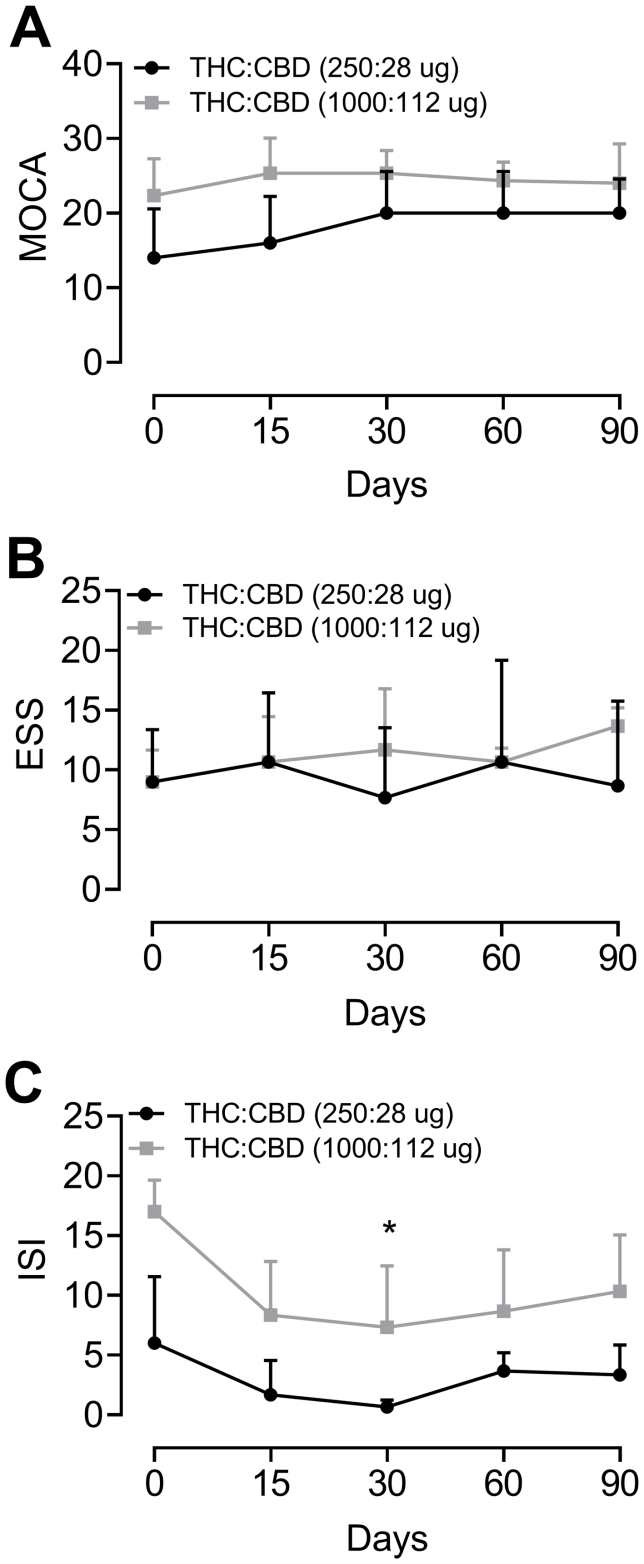
Cognitive evolution, daytime sleepiness, and insomnia in Parkinson’s disease patients before treatment (time 0) and undergoing treatment with Cannabis extract after 15, 30, 60, and 90 days of treatment. Evolution according to the scales: **(A)** Montreal Cognitive Assessment test (MoCA); **(B)** Epworth Sleepiness Scale (ESS); and **(C)** Insomnia Severity Index (ISI), respectively. Group 1 = THC:CBD (250:28 μg/day) and Group 2 = THC:CBD (1,000:112 μg/day). *p < 0.05 compared to basal data (Friedman test for repeated measures followed by Dunn’s post-hoc test).

For the statistical analysis of results, considering the limitation of the sample size, the data was treated as non-parametric. The Friedman test for repeated measures was applied, followed by Dunn’s post-hoc test to evaluate the individual response of each group relative to the baseline score. The profile of the participants in this study is presented in Table 1, while the results of the tests conducted are illustrated in Figure 2.

The statistical analysis indicated a significant benefit (*p* = 0.03) of the cannabis extract treatment, at dose of 1000:112 μg/day after 60 days of treatment, on insomnia assessed by ISI (Figure 2). Moreover, the statistical analysis of data from ISI and MoCA tests showed a trend toward improvement over time, while no significant effect was observed in the ESS.

The results of the MoCA evaluation showed that the patients in group 1 had lower basal scores compared to group 2. However, after the administration of the extract, the scores of the MoCA test increased, more markedly for patients in group 1, treated with THC:CBD (250:28 μg/day), as shown in Figure 2A. Nevertheless, statistical analysis indicated no significant difference. As for daytime sleepiness, there was no difference in ESS scores for both groups (Figure 2B). ISI evaluation results demonstrated that, overall, Cannabis extract treatment decreased the patients’ self-perception of difficulties in initiating or maintaining sleep compared to baseline values, for both groups. However, only patients from group 2 showed a statistically significant difference from baseline after 60 days of treatment (Figure 2C).

According to some patients interviews at the end of the trials, the use of cannabis oil helped them to sleep better. No self-reported of relevant side effects was mentioned by participants of this study. In a specific case, a patient who suffered for years with episodes of depression, weight loss, and sleep problems at night since PD diagnosis, described beneficial effects of cannabis extract treatment in his sleep, impacting positively his quality of life.

On the other hand, although the main aim of the current study was to investigate the impact of Cannabis extract treatment on non-motor symptoms, the PD patients reported improvement in their motor symptoms, especially listed in Table 1. Finally, all patients manifested interest in maintain the Cannabis extract treatment after this period of 90 days, since all them observed improvement in their PD symptoms.

## Discussion

The broad spectrum of actions of cannabinoids allows for targeting different aspects of many diseases. In fact, abnormal levels of endocannabinoids have been described in neurodegenerative diseases. For instance, the endogenous levels of anandamide and 2-arachidonoylglycerol (2-AG) are higher in PD patients than in healthy subjects (Murillo-Rodriguez et al., 2017). In this context, the administration of cannabinoids, particularly THC, may be beneficial in PD by modulating the altered endocannabinoid system in PD patients. As a partial agonist of CB1 and CB2 receptors, THC regulates the activity of this system, which presents abnormal levels of endocannabinoids, contributing to the regulation of neuroinflammatory and neuroprotective processes that are essential in managing PD (More and Choi, 2015).

Regarding the low doses of cannabinoids used in this study, the entourage effect suggests that the combination of different cannabis compounds, such as THC and CBD, can produce more effective therapeutic synergistic effects than the use of each compound isolated (Russo, 2011). This interaction allows lower doses of THC and CBD to be effective in treating conditions like PD, while minimizing the adverse effects commonly observed with higher doses of THC, such as cognitive impairment and anxiety, making the treatment safety for patients (Arnold et al., 2023).

The possible mechanisms by which the studied cannabinoids promote the therapeutic effects reported in this study primarily involve interaction with the endocannabinoid system, where THC acts on CB1 receptors in the central nervous system, promoting sedation and inducing sleep. CBD, in turn, indirectly modulates the activity of these receptors, in addition to possessing anxiolytic, antioxidant, and anti-inflammatory properties, which confer neuroprotection and enhance neuroplasticity, contributing to the preservation and potential improvement of cognitive function in neurodegenerative diseases such as PD (Suraev et al., 2020; Lotan et al., 2014; Crippa et al., 2019; Chagas et al., 2014).

Concerning the non-motor symptoms of PD focused in the current study, sleepiness and insomnia are associated with remarkably decline in several cognitive domains, including attention, working memory, executive functions, language, visuospatial skills, and episodic memory, impacting negatively the patients’ quality of life (Pfeiffer, 2016; Chaudhuri et al., 2006; Wallace et al., 2020). The treatment based on cannabis extracts provide different responses depending on many variables including individuals and schedule of administration (such as dose and duration). In fact, it has been highlighted that the biphasic effects of THC on cognitive processes are dose-dependent (Calabrese and Rubio-Casillas, 2018). In this context, high THC doses have been linked to cognitive impairment, while chronic administration of low THC doses improve cognitive performance of aging subjects (Calabrese and Rubio-Casillas, 2018; Bilkei-Gorzo et al., 2017). Additionally, we recently described original findings showing that microdoses of cannabis extract improved the cognitive performance of Alzheimer’s disease patients (Ruver-Martins et al., 2022).

In the current study, the cognitive performance of six PD patients assessed by MoCA scale was improved after cannabis administration during 90 days, at both tested doses. However, patients who received the lower dose (250:28 μg/day THC:CBD) presented a more marked improvement in comparison to baseline conditions. Moderate improvement was observed with the 1,000:112 μg/day THC:CBD dose, in which the patients expressed a mild effect. The observed improvement in PD patient scores can be explained by the actions of cannabinoids substances at different brain areas and mechanisms. The relationship between cannabis, cognition, and memory function has as a framework the expression of endocannabinoid system components in some brain areas, especially striatum, hippocampus, and prefrontal cortex (Crippa et al., 2019), in which has been established a high expression of the cannabinoid CB1 receptor, which underscores a critical role in the regulation of cognitive symptoms in PD. Indeed, Crippa and colleagues argued that the cognitive benefits associated to CBD treatment in PD underlying neurogenesis process in the hippocampus (Crippa et al., 2019).

THC use may be related to a memory decline in the next morning, while the administration of the association of THC:CBD (1:1) does not present this limitation, suggesting that the combination of these two phytocannabinoids, at specific doses, can attenuate side effects (Feeney et al., 2021). In this context, the extract used in the current study can be considered effective and safe, since the association of low doses of THC and CBD improved cognitive disturbance of six PD patients, while all participants no noticed negative effects of their THC/CBD treatment, but further investigation is warranted. Additionally, it is important to note that no adverse clinical effects were observed that could have resulted from any potential drug interactions. Table 1 displays the complete list of medications used to treat Parkinson’s disease.

Despite the use of THC in our study, even at low doses, it was expected that common adverse effects such as dry mouth, drowsiness, dizziness, increased appetite, and tachycardia could occur; however, none of these effects were observed, which is an important indication of the safety of this substance at utilized dosages. Nevertheless, the limitation of the sample size and the lack of a control group highlight the need for placebo-controlled clinical trials to confirm these findings.

Dopaminergic dysfunction associated with PD pathology also impact negatively the patients’ ability to compensate the sleep loss. This has a direct repercussion in sleep stages in PD, especially in insomnia disorders (Mantovani et al., 2018). Although clinical data related to cannabis administration in PD are limited, Souza and DiFrancisco-Donoghue (Souza and DiFrancisco-Donoghue, 2023) recently reporter that three PD patients of a case series study noted a subjective improvement in sleep after treatment with cannabis extract.

As can be appreciated in Figure 2C, there were significant changes in the patients who took the higher dose after 60 days of treatment. In this context, another study that supports these findings involved 1,793 adults with sleep disorders and reported adverse effects in 12% of the patients, with no serious effects observed, along with an improvement in sleep quality in more than half of the participants (Saleska et al., 2024).

Previous findings indicated that, regarding the general extract components, high doses of CBD (40 to 160 mg/day) increased the total sleep duration and improved sleep quality (Aarsland et al., 2021). A recent study also found an improved satisfaction with sleep quality with a dose of 300 milligrams of CBD, but no reduction in REM behavior disorder in PD patients (de Almeida et al., 2021). In the current study, four PD patients reported increased drowsiness after treatment with Cannabis extract, although no significant differences were found, as shown in Figure 2B. The trend toward increased daytime sleepiness may be related to higher doses and attributed primarily to the predominant and considerable proportion of THC in the extract, due to its nature as a sedative phytocannabinoid.

In conclusion, these results demonstrate a possible benefit of short-time treatment (3 months) with low doses of cannabis extract on cognition and insomnia in PD patients. This study provide novel findings of the potential of combining CBD and THC as safe and effective treatments for non-motor symptoms of PD. The limitations of this research include the small sample size, the absence of a placebo control group, and the lack of both preclinical and clinical studies supporting the therapeutic outcomes and safety of low-dose cannabinoids for the treatment of PD. Future work would include randomized clinical trials of CBD/THC treatment protocols and larger sample sizes of patients. This would allow for a more evidence-based approach to the utilization of CBD/THC in people with PD.

## Data Availability

The raw data supporting the conclusions of this article will be made available by the authors, without undue reservation.
